# Coexistence of contralateral cluster headache and probable paroxysmal hemicrania: a case report

**DOI:** 10.1186/s40064-016-2000-4

**Published:** 2016-03-31

**Authors:** Pauri Flavia, Lepre Chiara

**Affiliations:** Neurology Section, Department of Medico-Surgical Sciences and Biotechnologies, Sapienza University of Rome, Viale dell’Università 30, 00186 Rome, Italy

**Keywords:** Cluster headache, Paroxysmal hemicrania, Hypothalamus

## Abstract

**Introduction:**

The trigeminal autonomic cephalagias (TACs) are short-lasting unilateral headaches associated with autonomic features. Even if coexistence of different ipsilateral TACs in the same patient has been previously reported in few papers, the simultaneous occurrence of contralateral TACs is not described previously.

**Case description:**

A 50 years old working man complained, at the end of his cluster period, a new TAC, fitting the criteria for probable paroxysmal hemicrania. The dramatic improvement of this last cephalalgia with indomethacin treatment confirmed the diagnosis.

**Discussion, evaluation and conclusion:**

There is a clear overlap in clinical diagnosis between cluster headache (CH) and paroxysmal hemicrania (PH) and similarities are somewhat greater than differences. The originality of this report is the coexistence of contralateral TACs in the same patients at the same moment. According neuroimaging studies, CH hypothalamic activation occurs ipsilateral to the side of the headache while in PH hypothalamic activation occurs contralateral to the side of headache. It could be suggested that a continuous hypothalamic activation give a maladaptive plasticity recruiting closed neuronal aggregates responsible for the developing of PH after a long period of CH, confirming the central origin of both CH and PH. Further studies need to confirm this hypothesis.

## Introduction

The trigeminal autonomic cephalalgias (TACs: cluster headache CH, paroxysmal hemicrania PH, short lasting unilateral neuralgiform headache with conjunctival injection and tearing SUNCT, and hemicrania continua HC), are short-lasting unilateral headaches associated with autonomic features (Headache Classification Committee of the International Headache Society (IHS) 2013; Goadsby 2012; Martelletti 2015; Martelletti and Mitsikostas 2015). The coexistence of different ipsilateral TACs in the same patient has been previously reported in few published cases and the most common association was CH and PH (Tehindrazanarivelo et al. 1992; Centonze et al. 2000; Veloso et al. 2001; Fuad and Jones 2002; Shah and Prakash 2009; Totzeck et al. 2014): the two varieties of attacks seem to occur separately or simultaneously. The two disorders, therefore, might share some common pathophysiological aspects. We describe the unusual occurrence of two different contralateral TACs: cluster headache and probable paroxysmal hemicrania.

## Case description

FG, 50 years old working man came to our outpatient headache clinic complaining of recurrent attacks of headaches.

When he was 16 years old he suddenly begun to suffer of unilateral right orbital and sopraorbital severe pain, without radiation. He had usually two attacks per day, each lasting from 30 min to 2 h, every day at the same time: 2 or 3 o’clock a.m. and 2 or 3 o’clock p.m. Throbbing pain was associated with ipsilateral lacrimation, conjunctival injection, ptosis, nasal stuffiness. Restlessness or pacing activity was always present. Patient denied to experienced aura, nausea, photophobia or phonophobia. The attacks were concentrated in cluster lasting about 1 month, usually during summer, when he was young, and preferentially during winter in adult life. He remembered to have had about a cluster period every year in the last decade. He was treated with ergot, flunarizine, injectable sumatriptan.

The patient did not report a family history of headache. He smoked 30 cigarettes per day, drank about five cups of short-coffee per day, and some glasses of wine at dinner. During the pain periods alcohol intake triggered the beginning of the headache. He had suffered of gastric ulceration treated with proton pump inhibitor (omeprazole) and he was taking antihypertensive drugs.

The patient came to our clinic nearly at the end of the cluster period, but complained a new kind of headache, pulsating in quality, located on the left side, left temple, eye, and forehead, with radiation to the nostril associated with lacrimation, nasal congestion, photophobia, and phonophobia. He did not refer restlessness. These headache initially lasted 90 min, starting at 11 p.m., almost every night, rarely he had attack at 4 p.m. In the course of the time the attacks began more frequent: 4–5/day lasting about 30 min. The patient strongly asserted the new headaches were different from his usual previous ones. He was experienced these new headaches from 2 months and drugs he was using for cluster headache (verapamil, injectable sumatriptan) gave no relief, such as other nonsteroidal anti-inflammatory drugs (i.e. nimesulide, ergot). Routine blood tests revealed slight hyperglycemias and hypercholesterolemia. MRI of the head was normal.

Indomethacin, 50 mg taken at night, before sleeping, dramatically interrupted the crisis. The patient stopped the drug because of gastritis; this led to the recurrence of the headache. A this time Indomethacin 50 mg/daily was not enough, and the dose of 50 mg tid was gradually titrated together with increase in gastric protection. In 2 weeks the patient was again pain-free.

Indotest was not performed due to non availability of parenteral indomethacin (Antonaci et al. 1998) He was in indomethacin for 1 month after the last attack, and he is now headache free from 24 months. The patient gave his informed consent to the writing of this paper.

## Discussion and evaluation

According to the IHS classification (Headache Classification Committee of the International Headache Society (IHS) 2013) trigeminal autonomic cephalalgias (TACs) show the clinical symptoms of headache associated with signs of cranial autonomic system impairment: the presence of these signs made possible the diagnosis. TACs include Cluster Headache (CH) and Paroxysmal Headache (PH) sharing the same autonomic system impairment. There is a clear overlap in clinical diagnosis between CH and PH and similarities are somewhat greater than differences. The differential diagnosis actually points out the duration of headache—shorter in PH and longer in CH–, the frequency of the attacks—more frequent PH than CH–, and the response to indomethacin. The duration of CH is usually between 45 and 90 min, but briefer attacks, lasting 15 min have been described; on the other hand PH usually lasts 2–30 min, but 4 h attacks have been described too. The frequency is also different. In CH, headache could be present 3 times/week to 5–6/day; 5 attacks/day or more is the usual presentation in PH. Indomethacin is considered as a drug ineffective for CH patients whereas positive response is an essential criterion for the diagnosis of PH. At the same time some anedoctal reports are present in literature showing clinical efficacy of indomethacin in CH (Buzzi and Formisano 2003; Prakash et al. 2010).

The right sided headache complained by our patient fulfilled the criteria for CH, according the last International Classification of the Headache Society (Headache Classification Committee of the International Headache Society (IHS) 2013). Some doubts arise regarding the left-sided headache: it is actually a strictly unilateral headaches with autonomic features, i.e. lacrimation, nasal congestion; the pain is pulsating in quality, essentially over the temple and forehead. All these features fulfil the criteria for PH, or probable PH because according to the IHS classification (Headache Classification Committee of the International Headache Society (IHS) 2013) attacks of paroxysmal hemicrania occur in periods lasting 7 days to 1 year separated by pain-free periods lasting ≥1 month and we do not have till now the knowledge of a second attacks period, but the duration of the attacks is a little longer, and the frequency lower. On the other hand the sudden response to indomethacin, and the inefficacy of drugs previously used for CH, suggest the diagnosis of PH. Furthermore the patient clearly distinguished and differentiated the two kind of headaches and entered our clinic for the left sided headache, because the right sided headache was clearly recognized from him as the usual cluster headache he had suffered from a long time.

The simultaneous coexistence of CH and PH suggest the presence of some factors, both common and independent, which produce a condition that can give rise to both disorders. The two disorders, therefore, might share some pathophysiological aspects. Goadsby (2002) demonstrated that both paroxysmal hemicrania and cluster headache have raised calcitonin gene-related polypeptide and vasoactive intestinal polypeptide in the cranial circulation during attacks, suggesting a common pathogenesis. The finding that subcutaneous sumatriptan can work in CH and it could have partial efficacy in PH supports the hypothesis that these two TACs are closely related.

Activation of trigeminal autonomic reflex is considered essential for the manifestations of TACs, namely it is responsible for the acute attacks of TACs. The triggering factor for the activation of this reflex is probably situated in the hypothalamus. According neuroimaging studies, CH hypothalamic activation occurs ipsilateral to the side of the headache while in PH hypothalamic activation occurs contralateral to the side of headache (Holle et al. 2011). It is possible that the two conditions experienced by our patient share the activation of the strictly closed hypothalamic region. It could be possible that a continuous hypothalamic activation give a maladaptive plasticity recruiting closed neuronal aggregates responsible for the developing of PH after a long period of CH, but at this moment there are not data confirming this hypothesis. Actually the coexistence of different TACs in the same patients remains a rare phenomenon, however confirming that overlaps exist between CH and PH. Further studies need to explain the pathophysiology.

## Conclusion

In conclusion this is, at our knowledge, the first paper showing the unusual occurrence of two different contralateral TACs: cluster headache and paroxysmal hemicranias in the same patient and at the same time. Both these headaches share the activation of the strictly closed hypothalamic region. It could be possible that a continuous hypothalamic activation give a maladaptive plasticity recruiting closed neuronal aggregates responsible for the developing of PH after a long period of CH, even if there are now no data confirming this hypothesis. Further studies need to explain the pathophysiology.

## Abbreviations

TACs: trigeminal autonomic cephalalgias
CH: cluster headache
PH: paroxysmal hemicranias
